# Supplementary material for: A high-resolution mRNA expression time course of embryonic development in zebrafish
Source: eLife. 2017 Nov 16;6:e30860. doi: 10.7554/eLife.30860 (PMC5690287; doi:10.7554/eLife.30860)
Supplement: Supplementary file 6. [file elife-30860-supp6.zip › biolayout-clusters-files/Cluster195-genes.html]

Cluster195


# Cluster195: Genes

| Ensembl ID | Gene Name | Chr | Start | End | Biotype |
| --- | --- | --- | --- | --- | --- |
| ENSDARG00000090152 | anapc10 | 23 | 43916597 | 43922406 | protein\_coding |
| ENSDARG00000101561 | dbt | 22 | 16266801 | 16279551 | protein\_coding |
| ENSDARG00000013150 | dhx16 | 15 | 35041676 | 35075591 | protein\_coding |
| ENSDARG00000038695 | elavl1 | 2 | 37140876 | 37151741 | protein\_coding |
| ENSDARG00000057854 | sap18 | 9 | 21455815 | 21457707 | protein\_coding |
| ENSDARG00000038995 | tsnax | 13 | 48862684 | 48878999 | protein\_coding |
| ENSDARG00000039843 | znf800b | 4 | 3442338 | 3462052 | protein\_coding |
